# Effectiveness of a smartphone app on improving immunization of children in rural Sichuan Province, China: a cluster randomized controlled trial

**DOI:** 10.1186/s12889-016-3549-0

**Published:** 2016-08-31

**Authors:** Li Chen, Xiaozhen Du, Lin Zhang, Michelle Helena van Velthoven, Qiong Wu, Ruikan Yang, Ying Cao, Wei Wang, Lihui Xie, Xiuqin Rao, Yanfeng Zhang, Jeanne Catherine Koepsell

**Affiliations:** 1 Department of Integrated Early Childhood Development, Capital Institute of Pediatrics, No. 2 Yabao Road, Chaoyang District, Beijing, 100020 China; 2 Save the Children China Program, 1202 Block B Huaxi Buling, 5 Linyin Street, Wuhou District, Chengdu City, Sichuan Province China; 3 Global eHealth Unit, Department of Primary Care and Public Health, Imperial College London, Reynolds building 3rd floor, St Dunstans road, London, W68RP UK; 4 Save the Children China Program, 2-2-52 Jianwai Diplomatic Compound, Chaoyang District, Beijing, 100600 China; 5 Save the Children, 2000 L Street NW, Suite 500, Washington, DC 20036 USA

**Keywords:** Child immunization, Smartphone, Cluster randomized controlled study

## Abstract

**Background:**

The aim of this study was to assess the effectiveness of an EPI smartphone application (EPI app) on improving vaccination coverage in rural Sichuan Province, China.

**Methods:**

This matched-pair cluster randomized controlled study included 32 village doctors, matched in 16 pairs, and took place from 2013 to 2015. Village doctors in the intervention group used the EPI app and reminder text messages while village doctors in the control group used their usual procedures and text messages. The primary outcome was full vaccination coverage with all five vaccines (1 dose of BCG, 3 doses of hepatitis B, 3 doses of OPV, 3 doses of DPT and 1 dose of measles vaccine), and the secondary outcome was coverage with each dose of the five individual vaccines. We also conducted qualitative interviews with village doctors to understand perceptions on using the EPI app and how this changed their vaccination work.

**Results:**

The full vaccination coverage increased statistically significant from baseline to end-line in both the intervention (67 % [95 % CI:58-75 %] to 84 % [95 % CI:76-90 %], *P* = 0.028) and control group (71 % [95 % CI:62-79 %] to 82 % [95 % CI:74-88 %], *P* = 0.014). The intervention group had higher increase in full vaccination coverage from baseline to end-line compared to the control group (17 % vs 10 %), but this was not statistically significant (*P* = 0.164). Village doctors found it more convenient to use the EPI app to manage child vaccination and also reported saving time by looking up information of caregivers and contacting caregivers for overdue vaccinations quicker. However, village doctors found it hard to manage children who migrated out of the counties.

**Conclusions:**

This study showed that an app and text messages can be used by village doctors to improve full vaccination coverage, though no significant increase in vaccination coverage was found when assessing the effect of the app on its own. Village doctors using EPI app reported having improved their working efficiency of managing childhood vaccination. Future studies should be conducted to evaluate the impact of more integrated approach of mHealth intervention on child immunization.

**Trial registration:**

Chinese Clinical Trials Registry (ChiCTR): ChiCTR-TRC-13003960, registered on December 6, 2013.

**Electronic supplementary material:**

The online version of this article (doi:10.1186/s12889-016-3549-0) contains supplementary material, which is available to authorized users.

## Background

Immunization has significantly decreased the incidence of serious infectious diseases in infants and young children [1]. Childhood immunization is included in World Health Organization (WHO) and The United Nations Children’s Fund (UNICEF) Regional Child Survival Strategy as a major component of the child survival intervention package [2]. Therefore, child immunization is one of the essential child health strategies in primary care and is a critical public health objective. Although great achievement in childhood vaccination has been obtained worldwide, still there are children who do not receive on time. UNICEF reported that an estimated 83 % of children received three doses of diphtheria pertussis tetanus (DPT) combined vaccine and 84 % received their first dose of the measles vaccine [3]. In 2013, the WHO estimated that 1.49 million deaths in children less than 5 years of age could be prevented by immunization [4].

In China, implementation of the Expanded Program on Immunization (EPI) among children has considerably improved the national vaccination coverage. According to the fourth

National Health Service Survey (NHSS) in 2008, around 80 % of Chinese children aged 12-59 months were fully vaccinated (one dose of Bacillus Calmette Guéin (BCG) vaccine, measles vaccine (MV), three doses of hepatitis B vaccine (HBV), oral poliomyelitis vaccine (OPV) and DPT [5]. However, vaccination coverage varied greatly among provinces, with the highest coverage in Zhejiang Province (89.9 %), followed by Sichuan Province (70 %), Yunnan Province (70 %) and the lowest in Xinjiang Province (52 %). Thus, much work needs to be done in Chinese provinces with low vaccination rates to achieve the national target set for 2020, aiming that more than 95 % of Chinese children are fully immunized [6].

Information gaps for both the service-supplier and service-receiver (the population who need healthcare services) sides is seen as the main reason for children not receiving timely vaccination. In terms of the service supplier side, village doctors, who acquire the rural doctors practice license from government and practice medicine in rural areas, are in charge of childhood vaccination services in their catchment area. A study reported that the time interval between two injections was too long for doctors to remember the exact date for the next injection, which resulted in poor management and not actively tracking overdue children [7]. For the service-receiver side, some caregivers did not know where to get vaccination or which doctor was responsible for their child’s vaccination. As doctors did not inform caregivers about the date for next vaccination, children missed their vaccinations [7].

With the rapid development and use of information technology, mobile health (mHealth) interventions have been increasingly used. Mobile phones can be used to deliver interventions that can increase vaccination coverage [8]. Although more research on the effect, usability and cost on the effectiveness of vaccination interventions delivered by mobile phones has been carried out, studies using rigorous designs and reporting on robust evidence is still lacking [9]. Therefore, to improve vaccination coverage in rural Sichuan Province, an EPI smartphone application (EPI app) was developed and implemented in two townships in Xuanhan County, Dazhou City, Sichuan Province, China. The EPI app enabled village doctors who are in charge of child vaccination in their catchment area to keep vaccination records, track overdue children and learn about vaccination. The aim of this study was to assess the impact of the EPI app on improving child vaccination coverage in rural Sichuan Province.

## Methods

### Study design

**Fig. 1:**
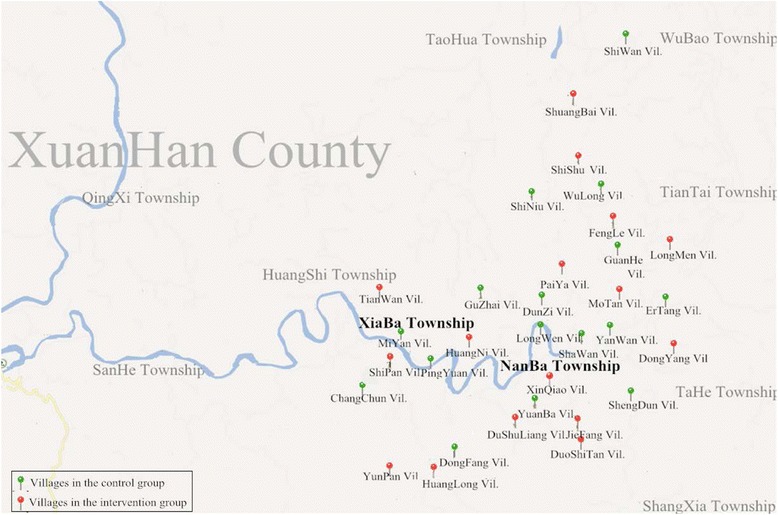
Maps of the studies villages

The protocol for this cluster randomized controlled trial was previously reported [10]. Village doctors were the unit of randomization and target of intervention (Fig.1). Village doctors were in charge of child vaccination (both management and injection) in their catchment area. There were in total 59 village doctors in our study area. Before randomization, we excluded 18 village doctors because there was more than one doctor in charge of vaccination in one village and we only maintained the youngest doctor in each village. We also excluded four village doctors because there were fewer than seven children under 2 years in their village, which meant that these villages were inadequate for our baseline and end-line survey. In addition, one village doctor was excluded because of paired study design.

A total of 36 village doctors were randomized: 18 were allocated to the intervention group and 18 to the control group. The village doctors were matched by three variables :1) the fraction of number of children aged under 1 year old divided by number of village doctors that were in charge of vaccination injection, 2) age of the youngest village doctor for vaccination in village, and 3) the population density (population/area in each village).

**Fig. 2:**
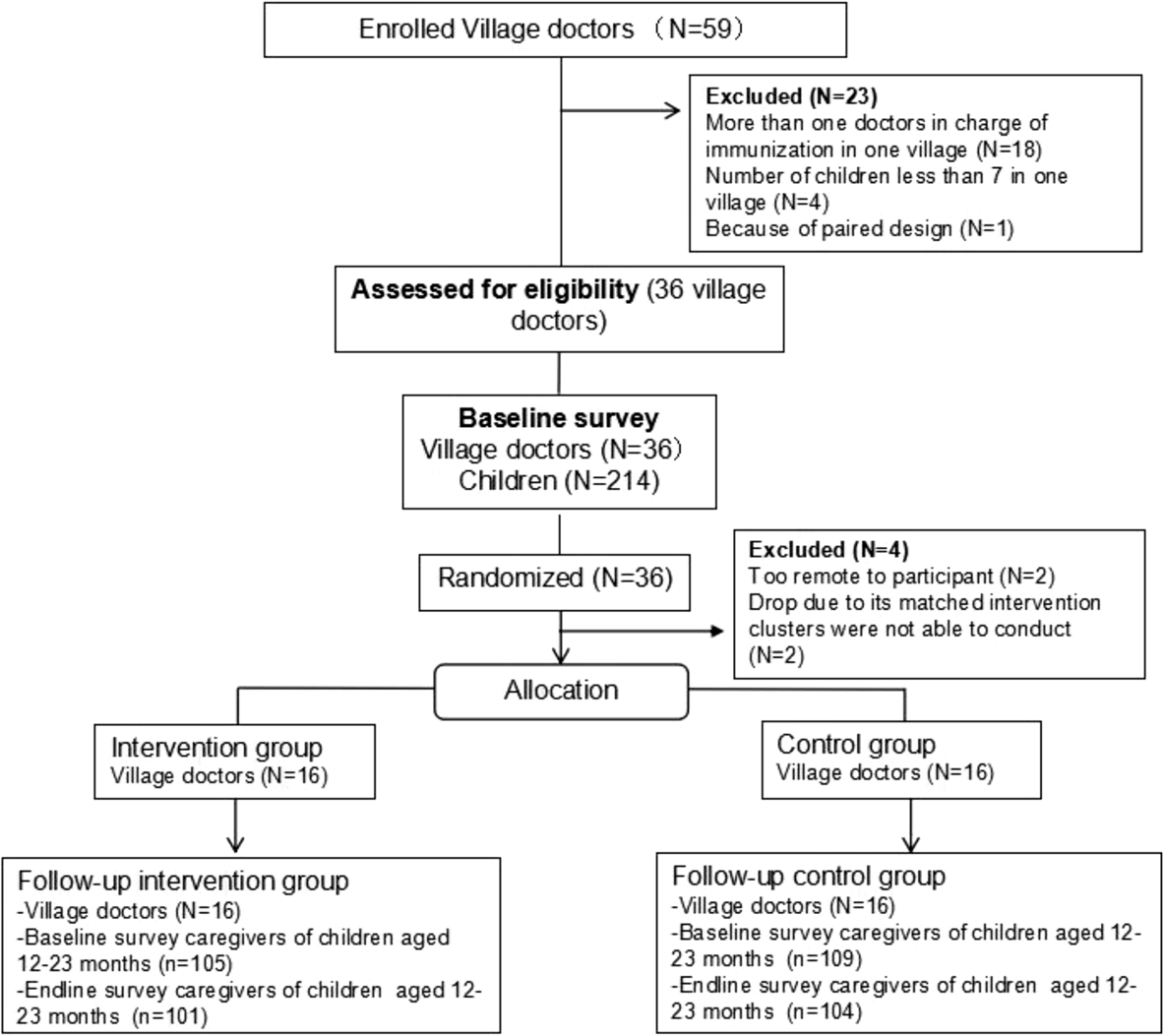
Flowchart of clusters and individual children allocated to intervention and control groups

We chose the block Tools package in the software programme “R” (https://www.r-project.org/) for matching and randomization. We created a measure between pairs of villages from several continuous variables (as we used the previously mentioned three variables). We set allowable range of differences between pairs of villages on one variable and then randomly assigned paired villages to intervention or control groups. After randomization, two villages in the intervention group were too remote to be reached and had to be excluded from the trial. Consequently, we had to exclude the two matched villages in the control group (Fig. 2). Therefore, there were a total 32 village doctors in our study with 16 of them in the intervention group and 16 in the control group.

### Study setting and population

This trial was conducted in two townships (Xiaba and Nanba township) in Xuanhan County in Dazhou City, Sichuan Province, China. Xuanhan County covers an area of 4271 km^2^ and has a total population of 1,170,000. It has 55 townships and 497 villages [11, 12].

### Intervention

In the intervention group, we gave a mobile phone with the EPI app to village doctors to manage child vaccination and we used text messages to alert caregivers about upcoming vaccinations. The EPI app had four modules with distinct functions: 1) making appointments; 2) recording vaccination status; 3) tracking overdue children; and 4) providing education.

The appointment and record modules were designed specifically for village doctors who vaccinated children so that they could record child vaccination status and upload data into the Child Immunization Register system at township level, which is the administrative level of village doctors. All village doctors in the intervention group used the tracking module to follow up overdue children. Overdue was defined as children who missed their scheduled vaccination appointment and did not receive the designated vaccination within three days after the scheduled appointment. Every day, the Child Immunization Register System identified overdue children and automatically sent the child’s name, missed vaccination, next appointment date and phone number of caregivers to the village doctor through the EPI app. That information was listed in the overdue functional module and village doctors could directly make a phone call to the caregivers of overdue children by tapping the phone number on the list of names. The program manager reimbursed RMB 30 (~5 USD) per month to each village doctor for fixed phone call minutes and a data plan for accessing the internet.

In the control group, village doctors conducted their vaccination work in their usual way and text messages were also implemented to alert caregivers about upcoming vaccinations. This was a change from our protocol because when we designed the study, the text message component was not included into the protocol. However, test messages were used because it may decrease the workload of village doctors. Shortly after the EPI app intervention was launched, we used the Child Immunization Register system to send text messages in both intervention and control groups at the same time and with the same intensity. The text messages included the appointment of vaccination time, date, recommended place and contact number of the local Center for Disease Control center, which is the administration institution for child vaccination. Thereby, village doctors did not need to make a notification before every scheduled vaccination appointment, but only track children who missed their vaccination appointment. At the end-line survey in January 2015, we sent a total of 28,566 text messages to caregivers of children.

### Outcome measures

#### Quantitative survey

Two cross-sectional household surveys were conducted at baseline (December 2013) and end-line (January 2015) to assess the vaccination coverage of children in rural Sichuan, China. The primary outcome was full vaccination coverage, defined as the percentage of surveyed children aged 12-23 months immunized with three doses of Hepatitis B, one dose of BCG, three doses of OPV, three doses of DPT and one dose of measles vaccine before their first birthday. The secondary outcomes were vaccination coverage indicators of one dose BCG, three doses of Hepatitis B, three doses of OPV, three doses of DPT, one dose of MV.

Based on a survey conducted in Xuanhan County, Sichuang Province in early 2013, the coverage of the full vaccination was 41.35 %. We expected that the full vaccination coverage in the intervention group would rise from 41.35 to 65 %. With a level of confidence of 95 % and power of 80 %, the coefficient of variation in the outcome between clusters within matched pairs (Km) as 0.25, [13] we estimated that 7 participants from each cluster of 18 pairs of clusters was sufficient to detect a statistically significant difference between the two groups. Since the aim of our study was to evaluate the effectiveness of the EPI app on the coverage of child vaccination, the change in the study design lowered the statistical power of the study (from originally 80 to 69 %), because the text messages in the control group could improve vaccination coverage [14].

We invited eligible caregivers of children aged 12-23 months who were living in Xuanhan County to participate in the household survey. Two modules (household information module and child immunization module) were selected from the Maternal, Newborn and Child Health Household Survey (MNCH HHS) Tool. The MNCH HHS was originally developed by WHO and then translated into Chinese in 2010 [15]. Trained interviewers administered structured questionnaires to collect general socioeconomic and vaccination information of children. Before the interview, caregivers were asked to bring the vaccination card with them if they had it. According to the survey protocol, if the caregiver brought a vaccination card for the child to the interview, the vaccination information (vaccine names, dates and places to receive the vaccinations) were copied exactly from the card. If the caregiver did not bring a vaccination card, a series of questions were asked to identify the vaccination status of BCG, OPV, DPT, Hepatitis B and MV with a “do not know” option for caregivers who could not remember accurately which vaccines the child had received

#### Qualitative interview

We conducted face-to-face interviews with village doctors at the end of the study. The interviews were designed to gain insight into perceptions of village doctors in the intervention group on using the EPI app. We also aimed to understand the routine way and the barriers of child vaccination management in the control group. A semi-structured interview guide was used to guide the interviewers. We used the purposive sampling and planned to interview all village doctors in the intervention (*n* = 16) and control groups (*n* = 16). However, we ended the interviews when no more new themes came up and thus saturation was achieved. Two research staff members (DXZ and XLH) conducted 29 semi-structured interviews (16 in the intervention group and 13 in the control group) at the end of the study. Each interview lasted about 20 min and was recorded using a digital recorder after we obtained the consent from village doctors.

### Randomization

After a bio-statistician generated the random allocation, the random allocation was send to the program manager at Save the Children, Sichuan Office, who installed the smartphone with EPI app and distributed this to village doctors in the intervention group.

### Data analysis

#### Quantitative

We used descriptive analyses to examine general characteristics of participants and vaccination coverage at individual level. We present the baseline and end-line level of primary outcome and the coverage of BCG, HBV1, HBV2, HBV3, OPV1, OPV2, OPV3, DPT1. DPT2. DPT3 and MV. The differences between baseline and end-line were compared using chi-square test and Fisher exact test. We calculated a summary statistic for each cluster. Then we compared the mean differences between the intervention and control clusters for each primary and secondary outcome using the paired t-test, which takes into account the matched-pair cluster design. If the difference in summary statistics between clusters was not normally distributed, the Wilcoxon signed ranks test was used rather than the paired t-test. We present the *P* value as an adjusted *P* value to reflect the transformation of the variable.

This analysis was different from the analysis described in our research protocol, in which random-effect logistic regression was planned to be used to test for the effect of controlling the covariate on individual level of on every outcome. We made the change for the following reasons: 1) the estimated within cluster variability was small; and 2) there were a relatively small number of matched clusters in our study. Where we could run the models, the paired t-test method tended to be conservative [13]. We used Stata Statistical Software: Release 13 (College Station, TX: StataCorp LP).

#### Qualitative

Two research staff members transcribed all interviews and discussions verbatim in Mandarin Chinese. A third research staff member checked the consistency of the transcripts and verified the transcripts by listening to the tapes. We used a thematic framework analysis to classify and organize the findings from the semi-structured interviews according to key themes [16]. Two researchers (CL and WQ) first independently read the transcripts to identify key themes. Then CL and WQ coded quotes, listed quotes related to our research question and organized the quotes into key themes in a table (in Mandarin). We used the tables to describe key themes and related beliefs and reasons. The two researchers discussed areas of agreement and discrepancies and further refined the coding scheme until consensus was reached on the findings and on the explanation from the analysis. Finally, CL translated the themes and related quotes into English and WQ reviewed the translated themes. We list all the key themes that we identified and related illustrative quotes.

## Results

### Quantitative survey

We interviewed 214 caregivers of children aged 12-23 months at baseline survey (105 in the intervention group and 109 in the control group) and 205 caregivers of children aged 12-23 months at the end-line survey (101 in the intervention group and 104 in the control group). Most interviewees were grandparents (53 % at baseline and 45 % at end-line) or mothers (37 % at baseline and 40 % at end-line). Around 80 % of our interviewed caregivers were the main caregivers of children (86 % at baseline and 79 % at end-line). Most of interviewees were Han Chinese (99 % both at baseline and end-line) and had an education level below high school (95 % at baseline and 92 % at end-line). The main source of the family income was work outside their hometown. Mobile phones were used by over 90 % of families. Children had a median age of 17-18 months and 54 % at baseline and 57 % at end-line were boys. Nearly 45 % of children were the first child of their mother. Most children (98 %) were delivered at a hospital and over 80 % of children were born full term (Table 1).

**Table 1:** General characteristics of surveyed caregivers and their children in rural Sichuan, China 2015 ^a^

	Baseline Survey (*N* = 214)^d^				End-line Survey (*N* = 205)^e^				*P* value (Baseline vs End-line)
	Total	Intervention	Control	*P* value	Total	Intervention	Control	*P* value	
	*n* (%)	*n* (%)	*n* (%)		*n* (%)	*n* (%)	*n* (%)		
Relationship to the child^b^				0.543				0.660	0.252
Mother	79(36.92)	36(33.03)	43(40.95)		83(40.49)	46(44.23)	37(36.63)		
Father	17(7.94)	9(8.26)	8(7.62)		26(12.68)	13(12.5)	13(12.87)		
Grandparents	114(53.27)	61(55.96)	53(50.48)		93(45.37)	44(42.31)	49(48.51)		
Others	4(1.87)	3(2.75)	1(0.95)		3(1.46)	1(0.96)	2(1.98)		
Main caregiver, yes	183(85.51)	93(85.32)	90(85.71)	0.935	161(78.54)	76(73.08)	85(84.16)	0.053	0.063
Source of family income									
Farming	128(59.81)	70(64.22)	58(55.24)	0.180	79(38.54)	37(35.58)	42(41.58)	0.377	<0.001
Work outside of hometown	166(77.57)	86(78.9)	80(76.19)	0.635	179(87.32)	89(85.58)	90(89.11)	0.447	0.009
self-employed	22(10.28)	7(6.42)	15(14.29)	0.058	15(7.32)	8(7.69)	7(6.93)	0.834	0.285
Others	25(11.68)	12(11.01)	13(12.38)	0.755	9(4.39)	5(4.81)	4(3.96)	0.767	0.006
Education level				0.999				0.566	0.171
Illiteracy	21(9.81)	11(10.09)	10(9.52)		30(14.63)	18(17.31)	12(11.88)		
Primary school	96(44.86)	49(44.95)	47(44.76)		97(47.32)	46(44.23)	51(50.50)		
Junior high school	85(39.72)	43(39.45)	42(40)		63(30.73)	31(29.81)	32(31.68)		
High School	12(5.61)	6(5.5)	6(5.71)		15(7.32)	9(8.65)	6(5.94)		
Ethnicity^b^				0.491				1.000	0.428
Han	213(99.53)	109(100)	104(99.05)		202(98.54)	102(98.08)	100(99.01)		
Other ethnicity minority	1(0.47)	0(0)	1(0.95)		3(1.47)	2(1.92)	1(0.99)		
Family communication device									
Mobile phone	203(94.86)	102(93.58)	101(96.19)	0.387	201(98.05)	102(98.08)	99(98.02)	0.976	0.079
Fixed-line telephone	39(18.22)	18(16.51)	21(20)	0.509	33(16.1)	13(12.5)	20(19.8)	0.155	0.564
Gender of children, boy	116(54.21)	54(49.54)	62(59.05)	0.163	116(56.59)	55(52.88)	61(60.4)	0.278	0.624
Age of children^c^, month	18.1(14.6-21)	18.1(15.1-21.8)	17.9(14.3-20.4)	0.307	17.2(14.6-19.7)	17.3(14.2-19.7)	17.0(14.8-19.5)	0.640	0.065
Gravidity				0.724				0.969	0.794
1	97(45.75)	52(48.6)	45(42.86)		87(44.39)	48(48.98)	39(39.8)		
2	88(41.51)	42(39.25)	46(43.81)		77(39.29)	37(37.76)	40(40.82)		
> =3	27(12.74)	13(12.15)	14(13.33)		32(16.33)	13(13.27)	19(19.39)		
Parity				0.567				0.059	0.477
primipara	98(45.79)	52(47.71)	46(43.81)		101(49.27)	58(55.77)	43(42.57)		
pluripara	116(54.21)	57(52.29)	59(56.19)		104(50.73)	46(44.23)	58(57.43)		
Gestational age, wk				0.111				0.397	0.072
Preterm	34(16.50)	12(11.54)	22(21.57)		18(19.57)	11(22.92)	7(15.91)		
Full term	172(83.50)	92(88.46)	80(78.43)		74(80.43)	37(77.08)	37(84.09)		
Deliver institution^b^				0.168				0.729	0.411
County or above hospital	110(51.4)	56(51.38)	54(51.43)		110(53.66)	55(52.88)	55(54.46)		
Township hospital	91(42.52)	43(39.45)	48(45.71)		78(38.05)	40(38.46)	38(37.62)		
Home	4(1.87)	4(3.67)	0(0)		2(0.98)	2(1.92)	0(0)		
Others	9(4.21)	6(5.5)	3(2.86)		15(7.32)	7(6.73)	8(7.92)		
Local rural hukou, yes	187(87.38)	99(90.83)	88(83.81)	0.122	189(92.2)	93(89.42)	96(95.05)	0.133	0.105

^a^ Chi-square tests were used unless otherwise specified. ^b^: Fisher exact test were used, ^c^: Wilcoxon rank-sum tests were used

^d^At baseline survey, 2 were missing for gravidity (2 in the intervention group) and 8 were missing for gestational age (5 in the intervention group and 3 in the control group)

^e^At end-line survey, 9 were missing for gravidity (6 in the control group, 3 in the intervention group), and 113 were missing for gestational age at birth(56 in the control group, 57 in the intervention group)

Table 2 shows the general profile of village doctors in two townships in Xuanhan County, Sichuan Province. The mean age of village doctors was 42 years. Village doctors were predominantly male (97 %) and all had Han ethnicity. Over 90 % of village doctors were certified rural doctors; 97 % were able to prescribe medicine; 56 % of them were at junior academic level and 86 % attended technical secondary school for medical education. Village doctors’ specialties were mainly clinical medicine (28 %) and western medicine and traditional Chinese medicine (TCM) combined (56 %). Apart for main family income, the basic characteristics of interviewees between baseline and end-line surveys were not statistically different. The general characteristics between the intervention and control groups were not significantly different.

**Table 2:** General characteristics of village doctors in Xuanhan, Sichuan China 2015 (*N* = 32)

Variables	Total (*N* = 32)		Intervention group (*N* = 16)		Control group (*n* = 16)		P**
	*n*	%	*n*	%	*n*	%	
Township							1.000
Xiaba	11	33.9	5	31.25	6	37.5	
Nanba	21	66.1	11	68.75	10	62.5	
Age,years*	42 ± 10.26		42.8 ± 9.88		42.13 ± 10.95		0.853
Gender							1.000
Male	31	96.88	15	93.75	16	100	
Female	1	3.13	1	6.25	0	0	
Marital Status							1.000
Unmarried	2	6.25	1	6.25	1	6.25	
Married	30	93.75	15	93.75	15	93.75	
Han Ethnicity	32	100	16	100	16	100	
Qualification for practice							
Certified rural doctors	29	90.63	15	93.75	14	87.5	1.000
Certificated physician	1	3.13	0	0	1	6.25	
Certificated assistant physician	1	3.13	0	0	1	6.25	
Certificated nurse practitioner	1	3.13	1	6.25	0	0	
Prescription privilege, Yes	30	96.77	15	100	15	93.75	1.000
Academic title							
Junior level	18	56.25	9	56.25	9	56.25	0.571
Intermediate level	8	25	5	31.25	3	18.75	
No academic title	6	18.75	2	12.5	4	25	
Basic education							
Junior high school	15	48.39	8	53.33	7	43.75	0.594
High School	16	51.61	7	46.67	9	56.25	
Professional education							
No professional education	1	3.13	1	6.25	0	0	
Technical secondary school/vocational high school	28	87.5	14	87.5	14	87.5	1.000
Junior college diploma	2	6.25	1	6.25	1	6.25	
Others	1	3.13	0	0	1	6.25	
Specialty							1.000
Clinical Medicine	9	28.13	7	43.75	2	12.5	
Western medicine & TCM	18	56.25	7	43.75	11	68.75	
Nurse	1	3.13	1	6.25	0	0	
Others	4	12.49	1	6.25	3	18.75	

*Presented as mean ± SD

**Fisher exact tests were used to compare the difference of proportion between intervention and control groups, except for age, where t-test was used

The full vaccination coverage (1 dose of BCG and MV, and 3 doses of HBV, DPT, OPV) was 69 % and at base-line coverage of BCG, 3 doses of HBV, DPT, OPV and MV were 97, 83, 84, 86 and 83 %, respectively (Table 3). At end-line, the full vaccination coverage was 83 %. Coverage of BCG, 3 doses of HBV, DPT, OPV and MV was 100, 93, 90, 93 and 93 %, respectively, and all increased between baseline and end-line survey. In intervention group, the coverage of full vaccination coverage, 3 doses of HBV, 3 doses of OPV and measles at end-line was 17, 8, 11 and 12 %, which were all statistically significantly higher than at baseline (*P* = 0.028, 0.031, 0.028, 0.001, respectively). In control group, the coverage of full vaccination coverage and 3 doses of HBV at end-line was 10 % and 12 %, and higher than at baseline (*P* = 0.014, 0.021, respectively).

**Table 3:** Vaccination coverage among surveyed children between baseline and end-line in intervention and control groups ^a^

	Intervention group			Control group			Total		
	Baseline (*n* = 105), % (95 % CI)	End-line (*n* = 101), % (95 % CI)	*P* value	Baseline (*n* = 109), % (95 % CI)	End-line (*n* = 104), % (95 % CI)	*P* value	Baseline (*n* = 214), % (95 % CI)	End-line (*n* = 205), % (95 % CI)	*P* value
BCG	97.3(92.2-99.1)	100.0(96.3-100.0)	0.247*	97.1(91.9-99.0)	99.0(94.8-99.8)	0.622*	97.2(94.0-98.7)	99.5(97.3-99.9)	0.122*
HBV1	99.1(95.0-99.8)	100.0(96.3-100.0)	0.122*	96.2(90.6-98.5)	100(96.5-100)	1.000*	97.7(94.6-99.0)	100(98.2-100)	0.061*
HBV2	95.4(89.7-98.0)	98.0(93.1-99.5)	0.171*	93.3(86.9-96.7)	97.1(91.9-99.0)	0.722*	94.4(90.5-96.8)	97.6(94.4-99.0)	0.100
HBV3	84.4(76.4-90.0)	92.1(85.1-95.9)	0.031	81.9(73.5-88.1)	94.2(88.1-97.4)	0.021	83.2(77.6-87.6)	93.2(88.9-95.9)	0.002
OPV1	97.3(92.2-99.1)	100.0(96.3-100.0)	0.163	98.1(93.3-99.5)	98.1(93.3-99.5)	0.689	97.7(94.6-99.0)	99.0(96.5-99.7)	0.277
OPV2	92.7(86.2-96.2)	97.0(91.6-99.0)	0.138	92.4(85.7-96.1)	96.2(90.6-98.5)	0.269	92.5(88.2-95.3)	96.6(93.1-98.3)	0.450
OPV3	85.3(77.5-90.8)	96.0(90.3-98.5)	0.028	87.6(80.0-92.6)	90.4(83.4-94.8)	0.259	86.5(81.2-90.4)	93.2(88.9-95.9)	0.023
DPT1	98.2(93.6-99.5)	98.0(93.1-99.5)	0.06	92.4(85.7-96.1)	95.2(89.3-98.0)	0.224	95.3(91.6-97.4)	96.6(93.1-98.3)	0.514*
DPT2	88.1(80.7-92.9)	95.1(88.9-97.9)	0.091	88.6(81.1-93.3)	92.3(85.7-96.1)	0.300	88.3(83.3-92.0)	93.7(89.5-96.3)	0.057
DPT3	83.5(75.4-89.3)	90.1(82.7-94.5)	0.181	83.8(75.6-89.6)	89.4(82.2-94.1)	0.207	83.6(78.1-88.0)	89.8(84.9-93.2)	0.066
MV	84.4(76.4-90.0)	96.0(90.3-98.5)	0.001	81.0(72.4-87.3)	90.4(83.4-94.8)	0.190	82.7(77.1-87.2)	93.2(88.9-95.9)	0.001
Five-vaccine immunization**	67.0(57.7-75.1)	84.2(75.8-90.0)	0.028	71.4(62.2-79.2)	81.7(73.5-88.1)	0.014	69.2(62.7-75.0)	82.9(77.2-87.5)	0.001

*BCG* Bacillus Chalmette Guerin vaccine, *HBV1,HBV2, HBV3* 1 or 2 or 3 doses of Hepatitis B vaccine, *DPT1 DPT2 DPT3* 1 or 2 or 3 doses of Pertussis Tetanus Combined vaccine, *OPV1 OPV2 OPV3* 1 or 2 or 3 doses of Oral Poliomyelitis Vaccine, *MV* Measles Vaccine

^a^ Chi-square tests were used to compare immunization coverage between baseline and end-line survey unless otherwise specified

*Fisher exact tests were used to compare immunization coverage between baseline and end-line survey

**Five-vaccine immunization indicates1 dose of BCG and measles, and 3 doses of HBV, DPT, OPV

**Fig. 3:**
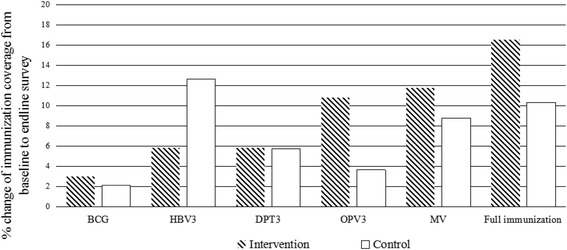
Immunization coverage change from baseline to endline survey at intervention and control groups

At baseline, the full vaccination coverage and the coverage of BCG, 3 doses of HBV, DPT, and OPV were not statistically significantly different between intervention and control groups. When comparing the difference from baseline to end-line survey between the intervention and control groups, the intervention group had a 17 % of increase in full vaccination coverage and the control group had a 10 % increase, but the differences between intervention and control groups was not statistically significant (*P* = 0.164). Similarly, vaccination coverage of BCG, DPT3, OPV3, measles vaccines were higher in the intervention group compared to control group, but the differences were not statistically significant (Fig. 3).

### Qualitative interviews

We found six themes in the qualitative interviews. Four themes (change of village doctors’ function, overdue vaccination tracking, management of migrant children, and health education) were reported by village doctors from both intervention and control groups. Two themes about perceptions of using EPI app (app usage and perceived benefits and barriers) were from village doctors in the intervention group.

#### Change of village doctors’ function

Village doctors in both the intervention and control groups mentioned that, up to recently, the Chinese government considered that village doctors may not be able to handle vaccine-related side effects. Therefore, the Chinese government shifted the main function of village doctors in managing child vaccination from vaccinating children to providing information about vaccination. As a result, very few village doctors vaccinated children in their catchment area (4/16 in the intervention group and 3/16 in the control group). Village doctors who vaccinated children said they only scheduled one vaccination day per month. However, as the government recently launched a catch-up vaccination campaign, some village doctors said they provided vaccination services during the campaign. Caregivers could choose to bring their children to township hospital or village clinics to receive vaccination. Thus, the main responsibility of village doctors regarding to child vaccination was now to track those children who missed their vaccination.

*Q: Do you provide vaccination service to the children in your catchment area?*

*“I used to provide vaccination services. It was four to five years ago. Now, I do not vaccinate children. The primary function now is to inform, go from door to door or make telephone calls to those overdue children, to remind caregivers to get vaccinations at township level.” (Village doctor, intervention village 7)*

*Q: Where did children in your catchment area receive vaccination?*

*“It depends on how far they live from the township hospital. They can choose to get their children vaccinated at my village clinic. Sometimes, they can also go to township hospitals. They can choose freely.....And in some cases, there is a catch-up vaccination campaign, then we need to vaccinate children in my village. For program vaccination, they all go to hospitals.” (Village doctor, intervention village 12)*

#### Overdue vaccination tracking

Before using the EPI app, the child vaccination information between township level and county level was not systematically integrated. Village doctors both in the intervention group and control group said they received a list of the names of children who missed their vaccination from the township hospital through various channels every month, including at monthly meeting or by telephone calls. Then village doctors were supposed to make phone calls or visit the children’s homes to remind caregivers about vaccination. However, this list only included the names of children, but no contact information, which made it hard for village doctors to identify and contact caregivers of those children.

*Q: How did you get the information of overdue children before using the EPI app?*

“Township hospital informed us. They made telephone calls and just said **(name of the child) missed **(certain type of vaccination), asked him/her to come at MM/DD (date). And then I went to inform caregivers. I did not inform them personally, but asked the village leader to remind those caregivers. (Because) there are more than 2000 people in my village, I cannot inform them (individually).”(Village doctor, intervention village 14)

Also obtaining information regarding overdue children was very time consuming and impractical.

*Q: How did you get the information of overdue children before using EPI app?*

“*I went to township hospital and got a list on paper. It took me half an hour to go back and forth. Sometimes more than an hour. It was inconvenient, because it took us a lot of time. And if villagers came to seek care, I was not available.”(Village doctor, intervention village 12)*

After using the EPI app, village doctors in the intervention group described the new work flow. The child immunization information system at township level automatically generated the list of names of children that were overdue and sent it to the EPI app in real time. Village doctors opened the EPI app and were able to obtain the list with detailed information including children’s name, parents’ name, telephone number and home address. This way, village doctors were able to make a phone call directly from the EPI app and track overdue children. If there was no telephone number or the number was wrong, village doctors could then make home visits according to the address on the name list from the EPI app. In addition, village doctors recorded the reason for being overdue and scheduled next vaccination dates using the EPI app. This information was uploaded to the township level information system.

*Q: After using EPI app, how do you track overdue children?*

*“Here (shows the screen to the interviewer). (This app) received information at the 7*^*th*^*of every month. See the names of those overdue children. If they did not get vaccination as appointed, then I record the reason for being overdue and click save. Then the reason for the vaccination being overdue is send to township. But before using the EPI app, we did not record reason for the vaccination being overdue.” (Village doctor, intervention village 15)*

#### Management of migrant children

Migrant children included children who moved out of the village (but were still permanent residents) and children who moved into the village (but were not permanent residents). For migrant children who moved out, village doctors reported that they did not manage their vaccination but continued to send reminder text messages and village doctors could continue managing both in the intervention and control groups. For village doctors in the intervention group, when migrant children who moved in and had received any vaccination in our project county, their vaccination history could be searched and downloaded from the EPI app. However, for migrant children who moved in but had not received any vaccination in our project county, village doctors would not manage those children’ vaccination because the vaccination history was unclear and this may result in the child to be vaccinated in multiple places or miss certain vaccine shots.

#### Health education

The education module in the EPI app aimed to let village doctors learn vaccination key knowledge and skills through the mobile phone conveniently. Compared to traditional on-site training, village doctors in the intervention group found this new module of continued education convenient and time-saving. However, the acceptability of this new way of education was mixed. Some village doctors said they preferred education through the EPI app; mostly because they could read information at any time, it was easy to retrieve information when they need it, it saved time to travel back and forth from the village to township hospital and they believed the information to be more comprehensive. However, some village doctors preferred traditional on-site training.

#### App usage

Most village doctors in the intervention group thought that the phone plan was sufficient for their expenses. However, when they needed to make a lot of long-distance phone calls (phone calls to other provinces) to track overdue children, costs of phone calls were higher. The reported frequency of app usage by village doctors in the intervention group varied extensively, ranging from once or twice per day to once or twice per month.

#### Perceived benefits and barriers

Village doctors in the intervention group perceived the EPI app to make their management of child immunization more convenient; mostly in getting the accurate list of names of overdue children with detailed information, reminding caregivers easily through phone calls and recording the reason for being overdue. Village doctors also reported that their management of child vaccination saved time (it was quicker to look up the contact information and make the phone call). With more comprehensive information, the need for village doctors to make home visit reduced because more reminders could be achieved through telephone calls. By recording the reason for being overdue, township level health workers could better understand why children did not receive vaccinations.

*Q: After using the EPI app, did this change your way of managing childhood vaccination? (If yes, in what way?)*

*“Well, it reduces my workload and saves my time. First in informing caregivers. Secondly in reporting information. And it also makes it easier to identify the contact information of caregivers who need to be reminded.” (Village doctor, intervention village 2)*

*“Well, it makes my work more convenient. (For overdue children) I just sit here and make telephone calls. This way, I do not need to walk to their home like before.” (Village doctor, intervention village 3)*

*“I believe it also benefited the township hospital (in managing child vaccination). Before, when we reported some information, it was just oral and may have caused some mistakes. But now we use EPI app to report. It is more accurate. The township hospital can gather more accurate information.” (Village doctor, intervention village 5)*

The major barrier for using the EPI app to manage child vaccination was that caregivers changed their mobile phone number. In rural Sichuan, many caregivers, especially parents, changed their number frequently. When this happened, village doctors needed to make a home visit and update the new mobile phone number. Another barrier for tracking children using the EPI app was that the home address for overdue children was not detailed enough. For village doctors who managed a village of over 1000 residents with several sub areas under one village, they preferred to list not only the village but also the sub area to help them identify the children. Internet access in some remote areas could be unstable and the system upgrade sometimes encountered problems. These factors also compromised usage of EPI app by village doctors. Only one village doctor reported that it was very hard for him to learn how to operate the EPI app and smartphone.

*Q: What do you perceive to be a major barrier for using the EPI app?*

*“From my point of view, the information is not detailed enough. For instance, our village has several sub areas. I only know children in my sub groups. If the name list of overdue children only includes address as my village but not the sub areas, it makes it very hard for me to track this child. I need to ask other people and I may not able to contact this child.” (Village doctor, intervention village 5)*

*“The biggest problem is when caregivers change their cell phone number. These caregivers change their mobile phone number quickly and did not inform me. So I have to make home visits and then update their new mobile phone number.”(Village doctor, intervention village 5)*

## Discussion

We used a cluster randomized controlled study design to assess the effectiveness of the EPI app on vaccination coverage. We also conducted qualitative interviews to understand how village doctors perceived childhood vaccination management. The full vaccination coverage and three doses of HBV increased significantly from baseline to end-line in both the intervention and control group. Although the intervention group had higher increase in vaccination coverage from baseline to end-line compared to the control group, the difference was not statistically significant. After using the EPI app, village doctors perceived their work of tracking overdue children to be more convenient and efficient.

To achieve universal coverage of child immunization, caregivers of children must have access to clinics where vaccination services are provided and be able to reach services when vaccinations are due [17]. This requires caregivers to be aware of when and where to bring their children to receive vaccination and actually go there. Furthermore, providers must be able to recognize when children are due for vaccination and deliver vaccination accordingly based on the vaccination program [17]. This requires the health care provider to actively track, vaccinate and follow up children who are due to be vaccinated and follow-up children who miss their vaccinations. Therefore, strategies to improve childhood vaccination ideally target to both service-receiver and service-supplier sides.

However, most previous studies focused only on the service receiver side or service supplier side. Text message reminders and reminders to parents, such as postcard reminder, computer-generated telephone reminders and reminder letters can increase child vaccination coverage [14, 18, 19]. Proactive follow-up of children overdue for vaccination by health care workers have shown to be a contributing factor in increasing vaccination coverage [20, 21].

Our study integrated interventions for both the service-receiver and service-supplier sides, which is thought to better facilitate children to get vaccination on time and to better manage overdue children. We found that groups with the EPI app plus text messaging as well as text messaging alone increased the coverage of full child vaccination. This is similar to a study in Zimbabwe that showed that when vaccination programs use multiple interventions, such as combining mail and phone reminders and case management home visits, is more effective than single interventions [17].

When evaluating the effect of EPI app alone by comparing the level of vaccination coverage change between intervention group and control group, the differences in vaccination coverage were not statistically significant. The limited impact of the EPI app may have been due to implementation of text messages in both the intervention and control group. Another possible reason is that maybe the vaccination coverage in our studied area was already relatively high. A similar result was reported from a study using a reminder system, integrated with provider reminders for overdue children, which led to relatively consistent but modest improvements in vaccination delivery to children [22]. A study in the US also reported that using specific reminders to children who were overdue for vaccination among children with delayed vaccination only improved catch-up vaccination to a small extend, which was not statistically significant [23].

Village doctors in the intervention group gave positive feedback on EPI app as the app supported their work to track overdue children could be done in a more convenient and timely manner. One challenge for village doctors to manage childhood vaccination was managing migrant children and children whose caregivers changed their cell phone numbers. Village doctors were unable to manage those children which may result in the child to be vaccinated in multiple places or miss certain vaccines. The difficulties of managing mobile population are not unique to rural China [24]. Caregivers needs aware of where and when to go for vaccination and the vaccination clinics who take care of migrant children also needs to get the vaccination history of those children [25]. One solution is facilitating integrated information sharing between vaccination clinics [25].

Our study has several strengths. The study took place in a real-life public health setting. We worked with village doctors within the existing health care structure. This means our study assessed the effectiveness of the intervention rather than its efficiency, which may facilitate replication and scale up. Also we combined qualitative and quantitative approaches, which gave us an opportunity to have a better understanding of whether and how the EPI app intervention had an impact on outcome measures.

There are also some limitations to our study. Firstly, we included younger village doctors when there was more than one doctor in a village. We made the assumption that they were better able to use smartphones. Therefore, the findings cannot be generalized for all health workers with a range of technology capabilities. Secondly, one year of implementation of the intervention was too short, because during the course of implementation, the EPI app was continuously modified to eliminate the system errors and improve the usability. By the time we conducted the end line survey, village doctors were still using EPI app, which will give us opportunity to conduct further study. Thirdly, four villages were excluded from our study due to two villages in intervention group were too remote to access. Also text messages were send to parents both in the intervention and control groups, which resulted in the estimated power to decrease from 80 to 69 %.

Future studies should be conducted to evaluate which components of mHealth interventions on child vaccination are most effective, such as the effectiveness of EPI app and text messaging. Also further studies need to assess the effectiveness of the EPI app in increasing coverage over a longer period of time and at a larger scale and to examine the impact of using a smartphone application on village doctors’ time to complete work, job satisfaction and their quality of care.

## Conclusions

We found that using the EPI app and text messaging reminders increased child vaccination coverage. However, EPI alone may not improve child vaccination coverage effective. Improved work efficiency of village doctors was also an important impact of the app.

## Additional file

Additional file 1: Raw database for EPI APP Program. (XLSX 21421 kb)

## Abbreviations

BCG: Bacillus calmette guerin vaccine
DPT: Diphtheria pertussis tetanus combined vaccine
HBV: Hepatitis B vaccine
HIB: Haemophilus influenzae type B vaccine
mHealth: Mobile health
MV: Measles vaccine
OPV: Oral poliomyelitis vaccine
ROT: Rotavirus vaccine
TCM: Traditional Chinese medicine
UNICEF: The United Nations Children’s Fund
WHO: World Health Organization
